# Immunomodulatory effects of hydatid antigens on mesenchymal stem cells: gene expression alterations and functional consequences

**DOI:** 10.3389/fmicb.2024.1381401

**Published:** 2024-04-09

**Authors:** Xin Wang, Wubulikasimu Mijiti, Zhifei Yi, Qiyu Jia, Junchao Ma, Zengru Xie

**Affiliations:** ^1^Department of Orthopedics and Trauma, The First Affiliated Hospital of Xinjiang Medical University, Ürümqi, Xinjiang, China; ^2^Key Laboratory of High Incidence Disease Research in Xingjiang (Xinjiang Medical University), Ministry of Education, Ürümqi, Xinjiang, China; ^3^Xinjiang Clinical Research Center for Orthopedics, Xinjiang Medical University, Ürümqi, Xinjiang, China

**Keywords:** mesenchymal stem cells (MSCs), hydatid disease, antigens, cytokine, immunomodulation

## Abstract

**Background:**

Cystic echinococcosis, caused by the larval stage of *Echinococcus granulosus*, remains a global health challenge. Mesenchymal stem cells (MSCs) are renowned for their regenerative and immunomodulatory properties. Given the parasite’s mode of establishment, we postulate that MSCs likely play a pivotal role in the interaction between the parasite and the host. This study aims to explore the response of MSCs to antigens derived from *Echinococcus granulosus*, the etiological agent of hydatid disease, with the hypothesis that exposure to these antigens may alter MSC function and impact the host’s immune response to the parasite.

**Methods:**

MSCs were isolated from mouse bone marrow and co-cultured with ESPs, HCF, or pLL antigens. We conducted high-throughput sequencing to examine changes in the MSCs’ mRNA expression profile. Additionally, cell cycle, migration, and secretory functions were assessed using various assays, including CCK8, flow cytometry, real-time PCR, Western blot, and ELISA.

**Results:**

Our analysis revealed that hydatid antigens significantly modulate the mRNA expression of genes related to cytokine and chemokine activity, impacting MSC proliferation, migration, and cytokine secretion. Specifically, there was a downregulation of chemokines (MCP-1, CXCL1) and pro-inflammatory cytokines (IL-6, NOS2/NO), alongside an upregulation of anti-inflammatory mediators (COX2/PGE2). Furthermore, all antigens reduced MSC migration, and significant alterations in cellular metabolism-related pathways were observed.

**Conclusion:**

Hydatid disease antigens induce a distinct immunomodulatory response in MSCs, characterized by a shift towards an anti-inflammatory phenotype and reduced cell migration. These changes may contribute to the parasite’s ability to evade host defenses and persist within the host, highlighting the complex interplay between MSCs and hydatid disease antigens. This study provides valuable insights into the pathophysiology of hydatid disease and may inform the development of novel therapeutic strategies.

## Background

Mesenchymal stem cells (MSCs) are multipotent cells derived from the mesoderm and are widely distributed in various tissues such as bone marrow, adipose tissue, and umbilical cord. They possess the ability to differentiate into osteoblasts, adipocytes, chondrocytes, and other cell types (Mollentze et al., 2021). In addition to their regenerative potential, recent research has indicated that MSCs also play a significant role in immunomodulation (Hu and Li, 2019). Through the release of a complex extracellular secretome containing multiple cytokines, chemokines, and extracellular vesicles (EVs), MSCs participate in the regulation of the inflammatory microenvironment and mediate interactions with other immune cells (Dabrowska et al., 2021).

Hydatid disease, also known as echinococcosis, is a zoonotic infectious disease caused by parasites belonging to the *Echinococcus* genus. One form of this parasitic infection, caused by *Echinococcus granulosus*, is referred to as cystic echinococcosis and can affect any part of the body (Brunetti et al., 2010). The cyst, a vesicle-like structure, is the characteristic focus of cystic echinococcosis and is found in the parenchymal organs of the host. Composed of materials produced by the parasite and host immune responses, the cysts have a multilayered structure (Gottstein et al., 2017). They contain various antigenic types and have been implicated in the modulation of the host immune response (Carmena et al., 2004; Kanan and Chain, 2006; Díaz et al., 2011).

In clinical practice, bone hydatid disease manifests when hydatid lesions infiltrate the bones, predominantly affecting the spine, pelvis, and femur, thereby significantly impairing patient health and functionality (Monge-Maillo et al., 2017). MSCs, known for their application in orthopedics and cardiovascular diseases, have shown potential therapeutic value through their immunomodulatory effects in parasitic infections, aiding in the efficacy of anthelmintic drugs via multiple pathways (Kian et al., 2022). Given that bone tissue harbors a rich supply of MSCs alongside diverse immune cells, previous studies have highlighted the close interaction between MSCs and immune cells (Fernández-Francos et al., 2021). Moreover, parasitic infections have been found to alter the gene expression profile of bone marrow cells (Zheng et al., 2021), with further research indicating the immunomodulatory capabilities of MSCs during such infections, potentially enhancing the effectiveness of antiparasitic treatments (Chulanetra and Chaicumpa, 2021; Su et al., 2023). Given the potential for hydatid disease foci to implant in various locations throughout the body, the widespread distribution of mesenchymal stem cells (MSCs) across tissues and organs similarly extends the significance of this research. The confluence of MSC distribution and hydatid lesion sites suggests that MSCs may demonstrate varied biological responses to different antigen types within the lesions, a factor that could influence the survival of the hydatid parasite within the host and the disease’s progression.

In this study, we characterized three major crude antigen types found in hydatid disease foci: excretory-secretory products (ESPs), hydatid cyst fluid (HCF), and particles from the laminated layer of *Echinococcus granulosus* (pLL), all of which are unpurified antigens comprising a diverse array of proteins and non-protein components (Maleki et al., 2023). Sheep are a common source of fertile cysts in studies on hydatid disease, while mice serve as a widely used model organism with a well-characterized genome. These antigens were co-cultured with mouse bone marrow-derived MSCs. High-throughput sequencing technology was employed to observe their impact on the mRNA expression profile of MSCs. Additionally, the secretory function of MSCs, as well as changes in cell cycle and cell migration under the stimulation of parasite antigens, were experimentally validated. These findings provide a reference for further investigation into the regulatory roles of MSCs in hydatid disease and the mechanisms of immune evasion.

## Materials and methods

### Collection and preparation of hydatid antigens

Sheep livers infected with *Echinococcus granulosus* cysts were sourced from Urumqi butcheries and transported to our laboratory under sterile conditions. Cysts were processed to extract the protoscolices-laden fluid, centrifuged, and washed. The purified protoscolices were cultured in α-MEM medium (Gibco) at 7500 protoscolices/ml and incubated for 24 h at 37°C in a 5% CO_2_ atmosphere. Post-incubation, the medium was centrifuged at 1000 × *g* for 10 min to harvest ESPs. C57BL/6 mice, provided by Xinjiang Medical University’s Laboratory Animal Center, were intraperitoneally injected with protoscolices. Six months later, mice were euthanized, and cystic fluid was collected post-cyst removal. The pLL was obtained by freeze-thawing the inner cyst walls, followed by mechanical disruption under a microscope. The lysate was centrifuged, and the supernatant containing pLL was filtered through a 0.22 μm filter (Millipore). Aliquots of ESPs, pLL, and HCF were stored at −80°C after supplementation with exosome-free fetal bovine serum (VivaCell) and antibiotics (HyClone).

### Isolation and cultivation of bone marrow MSCs (BMSCs)

BMSCs were isolated from 6- to 8-week-old C57BL/6 mice via the adherence screening method. Bones were processed to expose the marrow, which was then cultured in α-MEM with 10% fetal bovine serum and antibiotics at 37°C in 5% CO_2_. Adherent cells were expanded with media changes every 2 days until the third passage, which was used for flow cytometric analysis of MSC surface markers.

### Protein quantification, visualization, and cell proliferation detection

Protein quantification of hydatid antigens was performed using the BCA kit. Proteins were visualized as protein bands by dye-free polyacrylamide gel electrophoresis. Cell proliferation and activity were measured using the CCK8 assay. BMSCs were inoculated in 96-well plates and treated with ESPs, HCF, and pLL at different concentrations. CCK-8 solution was added and the absorbance at 450 nm was measured.

### Cell cycle and apoptosis detection

After 48-h co-culture with HCF, BMSCs were stained with Annexin V-FITC and PI and analyzed by flow cytometry for apoptosis rates. Cell cycle distribution was determined after 48-h co-culture by fixing cells with ice-cold 75% ethanol, RNase A treatment, and propidium iodide staining, analyzed using ModFit LT 3.2 software.

### Cell migration assay

Cell scratch assays assessed the migratory response of BMSCs to hydatid antigens, with quantitative analysis performed using Image J software.

### Sequencing analysis preparation

For transcriptomic analysis, BMSCs were treated with 2 μg/ml ESPs, HCF, or pLL for 48 h. Total RNA was subsequently extracted with TRIzol, quality-assessed, and used to construct cDNA libraries for Illumina NovaSeq sequencing. Differential expression analysis was conducted using Deseq2.

### Total RNA extraction and cDNA library construction

Total RNA was extracted, and its purity and concentration were assessed. mRNA was isolated and reverse transcribed to cDNA, which served as the template for library synthesis. The cDNA library underwent high-throughput sequencing on the Illumina NovaSeq platform.

### Bioinformatics and expression analysis

Differentially expressed genes (DEGs) were identified using Deseq2, and the clusterProfiler R package performed GO and KEGG enrichment analyses. The STRING database established PPI networks, and transcription factor interactions were examined using the TRRUST database and NetworkAnalyst.

### Real-time PCR validation

Five DEGs were selected for validation by quantitative PCR (JLM QX600). Total RNA was extracted from BMSCs and cDNA was reverse transcribed. qRT-PCR was performed using SYBR Green Master Mix, and the ΔCt method was used to calculate the relative expression of target genes. The primer sequences used in PCR experiments are listed in Table 1.

**Table 1:** Primer sequences used in qRT-PCR.

Genes	Forward (5′-3′)	Reverse (5′-3′)
NOS2	ATCTTGGAGCGAGTTGTGGATTGTC	TAGGTGAGGGCTTGGCTGAGTG
COX2	TGGTGCCTGGTCTGATGATGTATG	GTCTGCTGGTTTGGAATAGTTGCTC
MMP3	ACGATGATGAACGATGGACAGAGG	GCCTTGGCTGAGTGGTAGAGTC
IL-6	CTCCCAACAGACCTGTCTATAC	CCATTGCACAACTCTTTTCTCA
MCP-1	ACTCACCTGCTGCTACTCATTCAC	TTCTTTGGGACACCTGCTGCTG
CXCL1	TGGCTGGGATTCACCTCAAGAAC	GTGTGGCTATGACTTCGGTTTGG

### Western blots test

Protein lysates were prepared from BMSCs and separated by SDS-PAGE electrophoresis. The proteins were transferred to a PVDF membrane and blocked with skimmed milk. Primary and secondary antibodies were added, and immunoreactivity was detected using chemiluminescence. The relative expression level of the target protein was analyzed using ImageLab software.

### Enzyme linked immunosorbent assay and nitric oxide concentration detection

Cell supernatants were collected from BMSCs and subjected to specific assays to measure the concentrations of cytokines and nitric oxide (NO). ELISA was performed to detect the concentration levels of MCP-1, TNF-α, IL-6, PGE2, and IL-4 using commercially available ELISA kits. In addition, the NO content in the cell supernatant was measured using a NO colorimetric test kit.

### Statistical analysis

The data in this study were analyzed and visualized using GraphPad Prism software (version 8.0). Measurement data were presented as mean ± standard deviation. An independent sample t-test was employed for comparing two groups, while one-way analysis of variance was used for comparing multiple groups. Statistical significance was denoted as *p < 0.05, **p < 0.01, ***p < 0.001, and ****p < 0.0001.

## Results

### Protein profiles and concentrations of hydatid antigens

**Figure 1:**
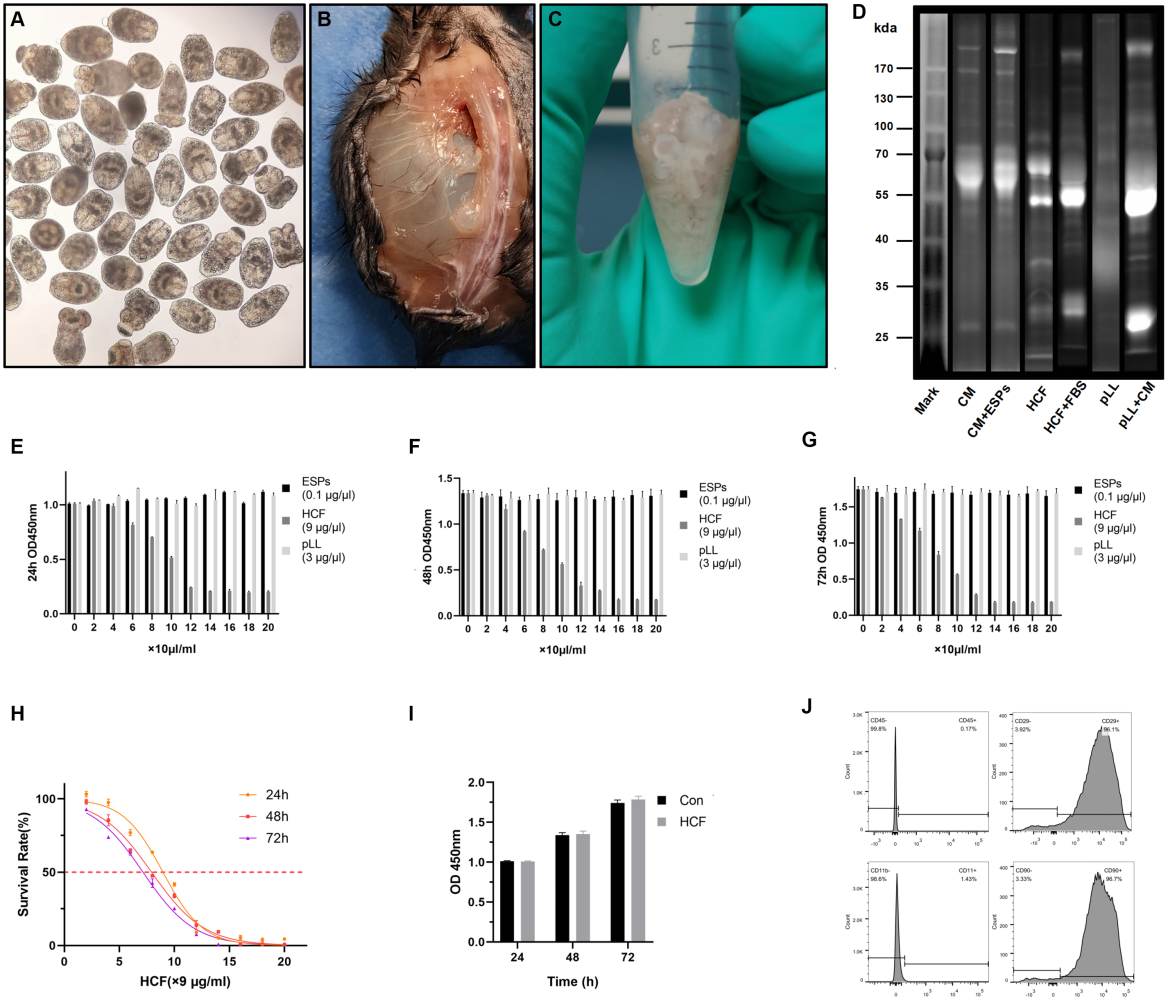
Extraction of hydatid and cell samples and their effect on the proliferation of BMSCs. **(A)** Extracted Protoscolex of *Echinococcus granulosus*. **(B)** Parasitized hydatid cysts in the paraspinal and abdominal cavities of mice. **(C)** Hydatid cysts used to extract pll after extraction of cyst fluid. **(D)** Distribution of bands of hydatid antigenic proteins on stain-free gel. CM, complete medium; ESPs, excretory-secretory products; HCF, hydatid cyst fluid; FBS, fetal bovine serum; pLL, particles from the *Echinococcus granulosus* laminated layer. **(E–G)** Absorbance at 450 nm after treated BMSCs with three different concentrations of hydatid antigen for 24–72 h in CCK8 experiments. **(H)** Survival rates of BMSCs cells treated with different concentrations of HCF over 24–72 h. **(I)** Absorbance at 450 nm of control and 7.8 μg/ml HCF-treated BMSCs for 24-72 h in CCK8 experiments. **(J)** Expression of the surface antigens CD45, CD29, CD11b, and CD90 on extracted mouse bone marrow-derived MSCs as provided by flow cytometry. The data are presented as the mean ± SD. *p < 0.05, **p < 0.01, ***p < 0.001, significantly different from control group.

The Protoscolex, ESPs, HCF and pLL were extracted from the liver of affected sheep and the intraperitoneal cavity of C57BL/6 mice (Figures 1A–C). BCA protein assays revealed that 24-h ESPs from 7,500/ml protoscolices yielded approximately 10 μg/ml, while mouse intraperitoneal HCF and pLL averaged 900 μg/ml and 300 μg/ml, respectively. SDS-PAGE analysis indicated protein molecular weights for ESPs (55–180 kDa), HCF (40–100 kDa), and pLL (25–135 kDa) (Figure 1D).

### BMSCs phenotypic confirmation

Through flow cytometry analysis, the phenotype of BMSCs was confirmed, revealing expression levels of CD29 (96.1%) and CD90 (96.7%), while negative markers CD45 and CD11b were minimally expressed at 0.17 and 1.43%, respectively (Figure 1J).

### Influence of hydatid antigens on BMSCs proliferation

The proliferation assays demonstrated that ESPs (0–2 μg/ml) and pLL (0–60 μg/ml) had a negligible impact on BMSCs proliferation across all tested time points. Conversely, HCF showed IC50 values of 80.6, 71.6, and 64.2 μg/ml at 24, 48, and 72 h, respectively, indicating a dose-dependent inhibitory effect on cell proliferation (Figures 1E–I).

### Effects of hydatid antigens on BMSCs apoptosis and cell cycle

**Figure 2:**
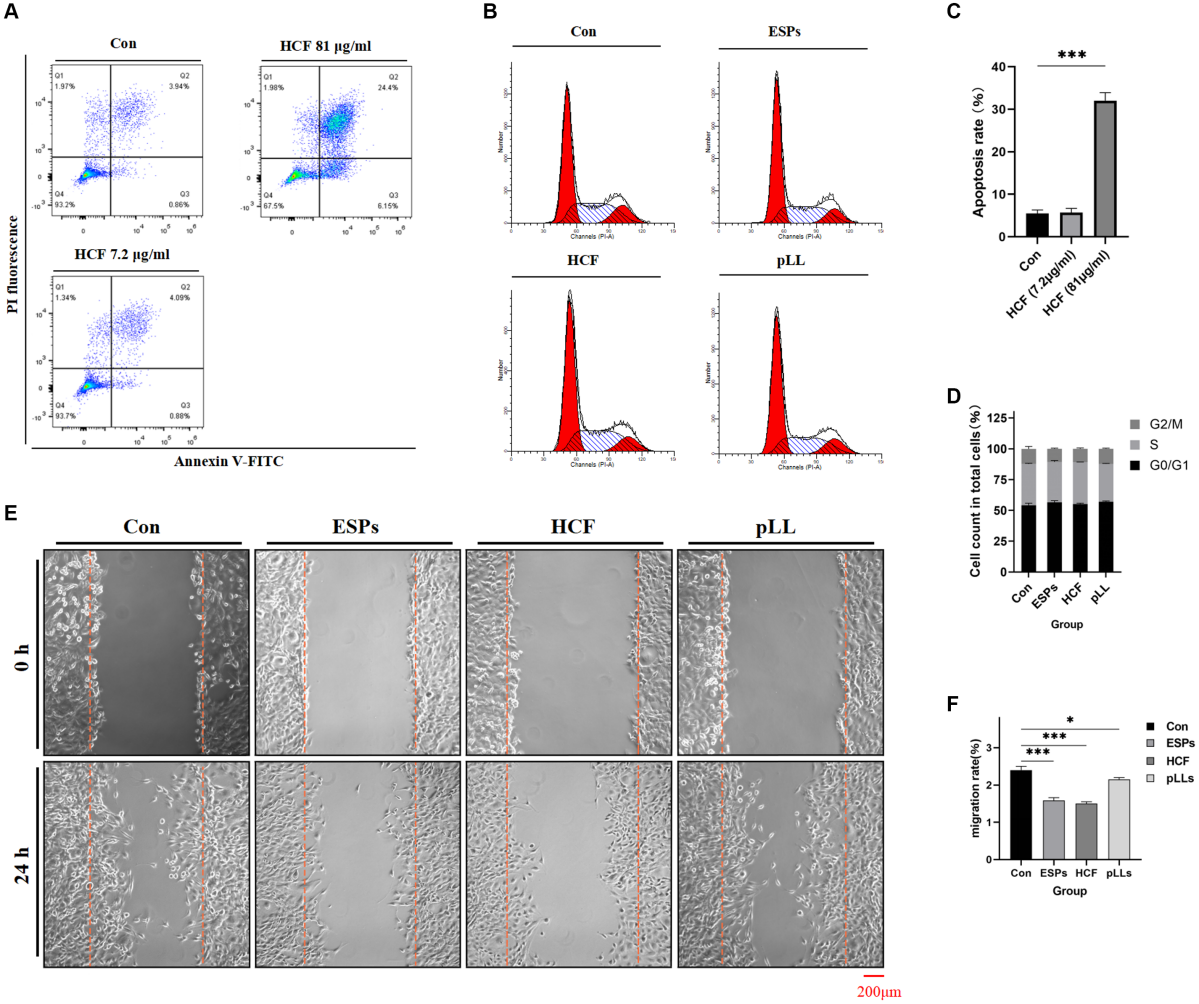
Apoptosis, cycle and migration of BMSCs in each group. **(A)** Apoptosis of BMSCs after treatment with different concentrations of HCF provided by flow cytometry. **(B)** Cell cycle distribution of the four groups. **(C)** Comparison of apoptosis rates of the three groups. **(D)** Cell cycle distribution of the four groups presented by stacked bar graphs. **(E)** 0–24 h cell scratch experiments. **(F)** Cell migration rate of the different groups counted in the scratch experiments and the differences between groups. The data are presented as the mean ± SD. *p < 0.05, **p < 0.01, ***p < 0.001, significantly different from control group.

Exposure to HCF at 7.2 μg/ml did not significantly alter apoptosis rates in BMSCs. However, a concentration of 81 μg/ml was associated with an increase in apoptosis rates (Figures 2A,C). Cell cycle analysis across control, ESPs, HCF, and pLL groups revealed no significant differences, suggesting that these antigens do not markedly affect cell cycle progression in BMSCs (Figures 2B,D).

### Differential gene expression analysis

**Figure 3:**
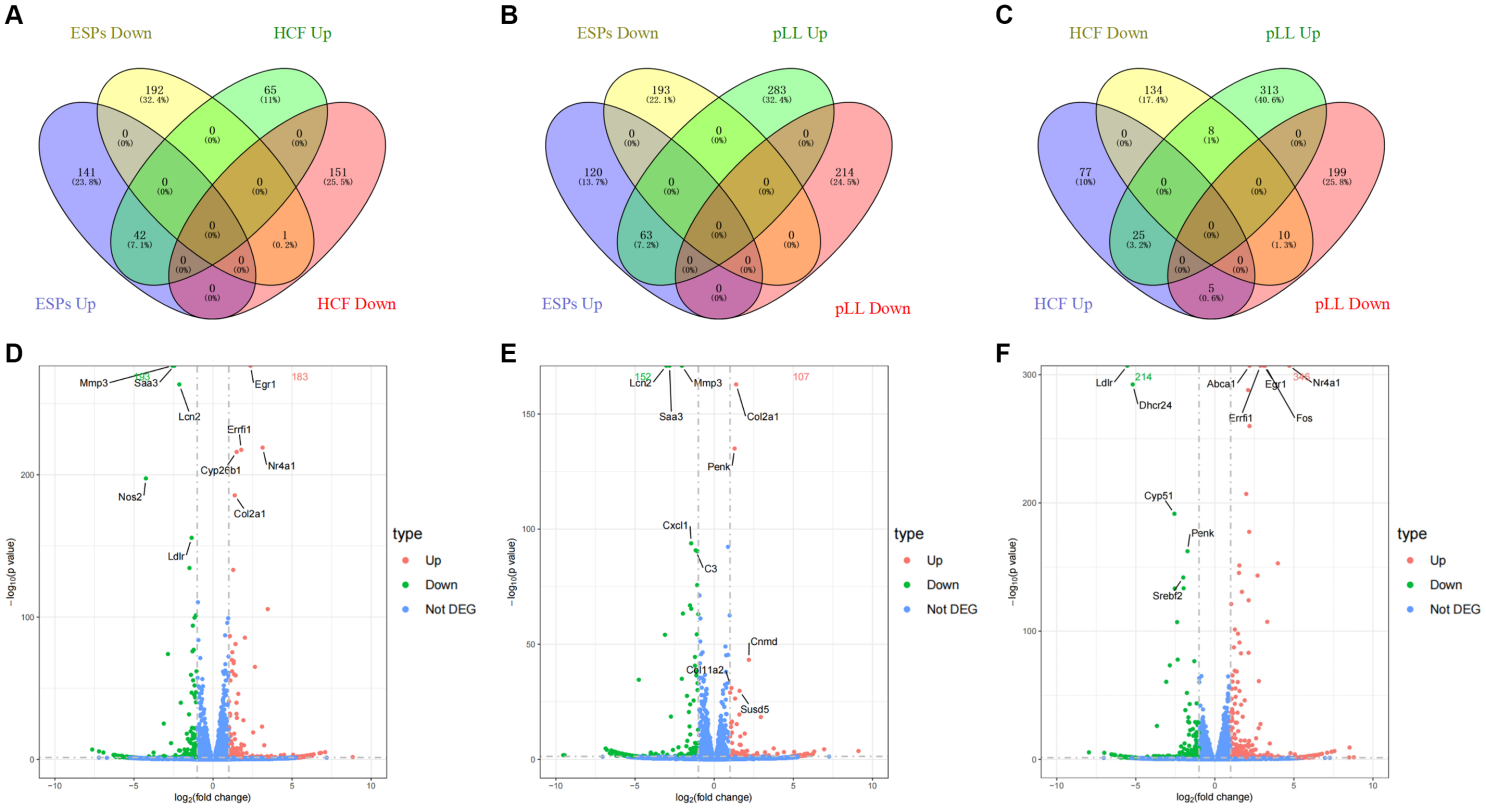
Distribution of differentially expressed genes. **(A–C)** Venn diagram two-by-two comparison of three groups of differentially expressed genes grouped according to up-/down-regulation and showing the number and proportion of overlapping genes. **(D)** Volcano plot of differentially expressed genes in the ESPs group. **(E)** Volcano plot of differentially expressed genes in the HCF group. **(F)** Volcano plot of differentially expressed genes in the pLL group.

After 48-h antigen co-culture, notable gene expression changes were observed: 376 genes in ESPs group (183 up, 193 down), 259 in HCF group (107 up, 152 down), and 560 in pLL group (346 up, 214 down). Common up- and down-regulated genes were identified between groups (Figure 3 and Table 2), indicating significant and distinct effects of different echinococcal antigens on gene transcription in BMSCs.

**Table 2:** Top 10 genes in the three groups with differential expression.

Group	Gene ID	baseMean	log2FC	adj.p.Value	Type
ESP/Control	Gm49601	121.511	10.360	0.045	Up
	Krt6b	42.152	8.833	0.104	Up
	Krt16	100.407	5.488	0.093	Up
	Egr3	849.406	3.460	0.000	Up
	Nr4a3	63.130	3.237	0.000	Up
	Nr4a1	2900.618	3.137	0.000	Up
	Hapln1	276.004	3.098	0.000	Up
	Krt1	208.927	2.956	0.049	Up
	Egr2	697.165	2.653	0.000	Up
	Eid3	280.298	2.540	0.000	Up
	Nos2	2475.917	−4.248	0.000	Down
	Scd3	35.323	−3.760	0.000	Down
	Saa2	204.533	−3.116	0.000	Down
	Saa1	784.297	−2.852	0.000	Down
	Mmp10	99.850	−2.655	0.000	Down
	Mmp3	60042.417	−2.557	0.000	Down
	Saa3	134519.037	−2.447	0.000	Down
	Tnfrsf9	27.032	−2.369	0.008	Down
	Vnn3	66.181	−2.148	0.000	Down
	Lcn2	55866.798	−2.133	0.000	Down
HCF/Control	Krt6b	50.525	9.115	0.007	Up
	Gpr20	11.257	6.948	0.001	Up
	Nppa	7.277	6.319	0.010	Up
	Paqr6	11.086	5.051	0.014	Up
	Stmn4	14.310	4.408	0.016	Up
	Calca	28.547	3.762	0.001	Up
	Chdh	20.550	3.209	0.019	Up
	Hapln1	247.747	2.947	0.000	Up
	Krt6a	59.854	2.709	0.036	Up
	Cnmd	709.598	2.193	0.000	Up
	Mucl3	10.734	−6.859	0.001	Down
	Nmur1	10.172	−6.782	0.002	Down
	Zglp1	8.918	−6.591	0.038	Down
	1700016H13Rik	8.388	−6.504	0.004	Down
	Txndc2	7.942	−6.424	0.011	Down
	Cyp2j9	7.669	−6.375	0.008	Down
	Minar1	7.014	−6.244	0.014	Down
	Krtap6-5	6.199	−6.068	0.029	Down
	Trhde	5.947	−6.007	0.035	Down
	Chrna2	5.257	−5.830	0.050	Down
pll/Control	Prnd	31.725	8.523	0.000	Up
	Egr4	16.314	7.563	0.000	Up
	Knl1	14.395	7.382	0.000	Up
	Sec1	11.893	7.105	0.000	Up
	Dpp6	9.487	6.778	0.001	Up
	Fibcd1	8.403	6.607	0.002	Up
	Crmp1	8.088	6.553	0.003	Up
	Gpr61	7.333	6.404	0.006	Up
	Rpp25	7.126	6.368	0.004	Up
	Lypd1	6.551	6.246	0.042	Up
	Gm49368	1144.477	−7.966	0.000	Down
	Plek2	12.435	−6.996	0.000	Down
	Mag	9.096	−6.545	0.002	Down
	Htr1d	8.520	−6.449	0.002	Down
	B3gnt5	7.890	−6.339	0.004	Down
	Ly6g6d	7.690	−6.303	0.005	Down
	Nxpe2	6.241	−6.002	0.019	Down
	Spn	6.108	−5.970	0.012	Down
	Lce1a1	5.848	−5.906	0.019	Down
	Clec2h	5.042	−5.693	0.036	Down

### GO and KEGG pathway analysis of DEGs

**Figure 4:**
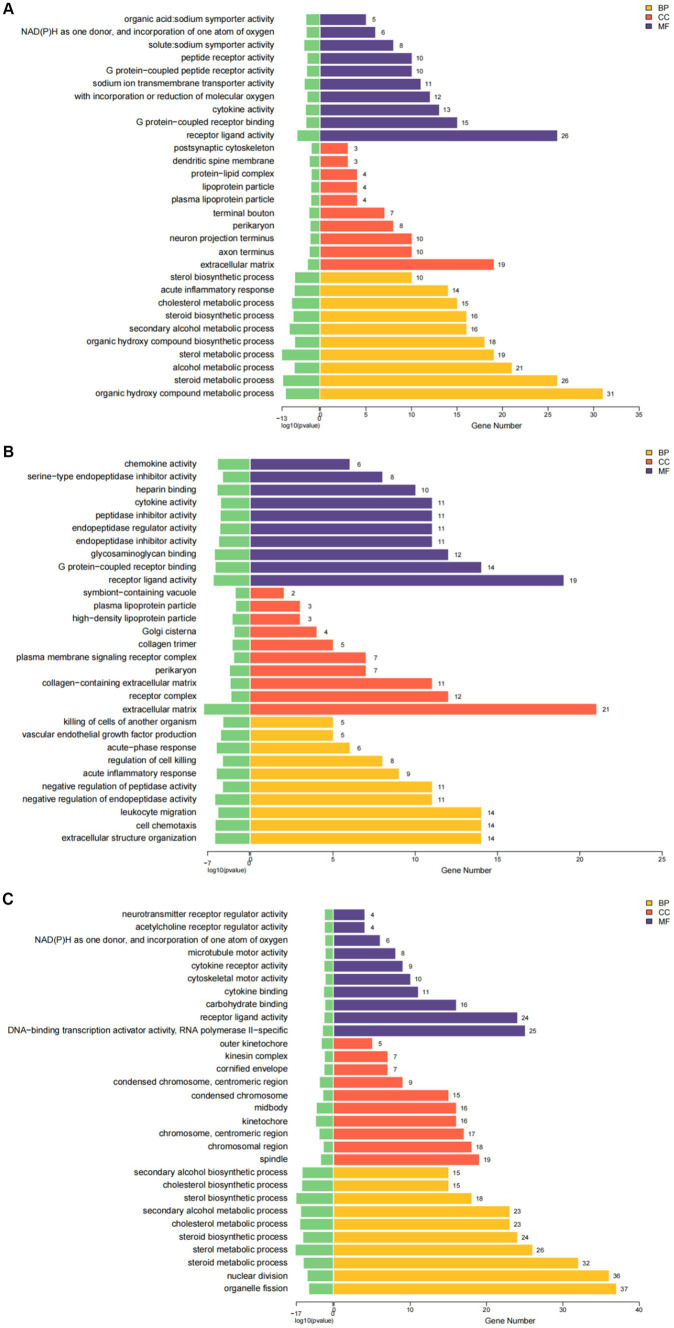
The results of GO enrichment analysis of differential genes are presented as histogram graphs. **(A)** GO enrichment analysis of the ESPs group. **(B)** GO enrichment analysis of the HCF group. **(C)** GO enrichment analysis of the pLL group.

**Figure 5:**
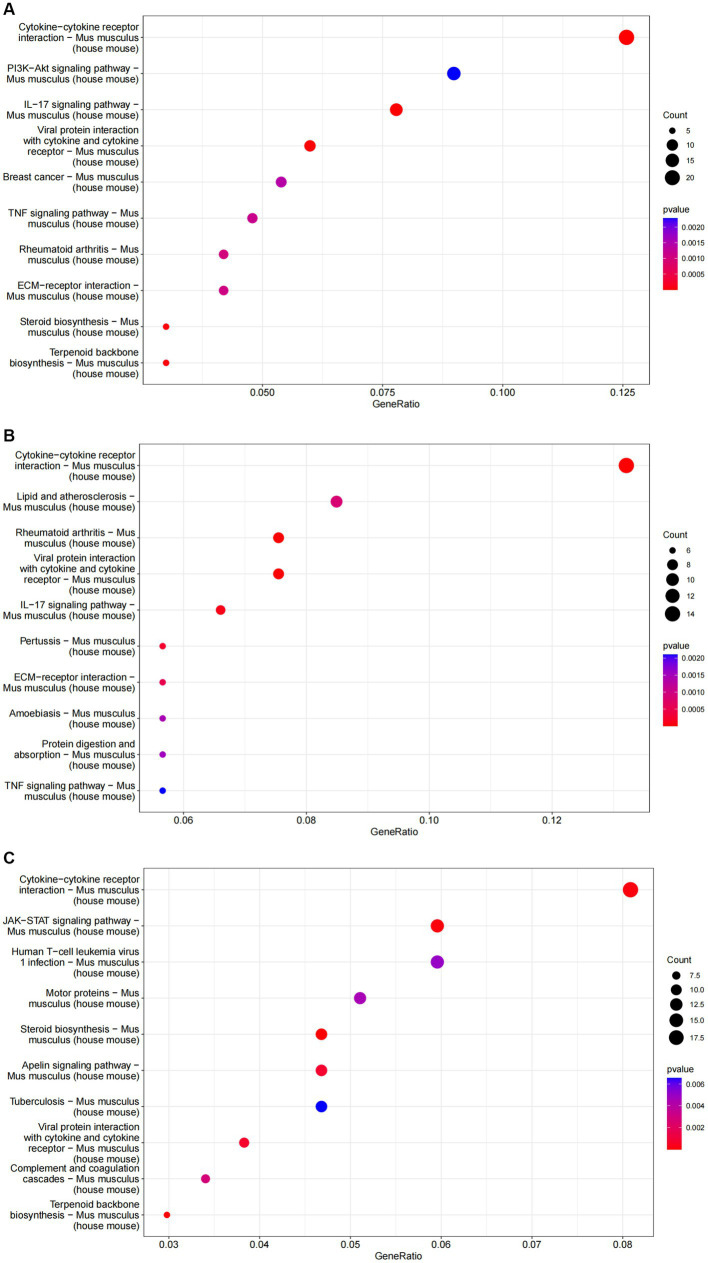
The results of KEGG enrichment analysis of differential genes are represented as bubble plots. **(A)** KEGG enrichment analysis of the ESPs group. **(B)** KEGG enrichment analysis of the HCF group. **(C)** KEGG enrichment analysis of the pLL group.

GO analysis highlighted that DEGs were primarily enriched in molecular functions such as cytokine activity, chemokine activity, and oxidoreductase activity; cellular components like the extracellular matrix and receptor complex; and biological processes including cell chemotaxis and leukocyte migration. KEGG pathway analysis revealed involvement in cytokine-cytok receptor interaction, TNF signaling pathway, and IL-17 signaling pathway, indicating the antigens’ impact on immune response pathways (Figures 4, 5).

### Protein–protein interaction network analysis

**Figure 6:**
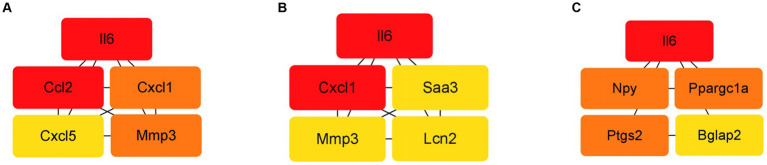
Top 5 HUB genes in each group in the PPI network. **(A)** Top 5 HUB genes in the ESPs group. **(B)** Top 5 HUB genes in the HCF group. **(C)** Top 5 HUB genes in the pLL group.

The protein–protein interaction (PPI) network analysis identified key hub genes (IL-6, Ccl2, Cxcl1, Cxcl5, Mmp3) and transcription factor-target gene interactions, suggesting these genes play central roles in the response of BMSCs to hydatid antigens. This analysis underscores the complexity of the immunomodulatory effects exerted by the antigens on BMSCs (Figure 6 and Table 3).

**Table 3:** TF genes and their overlapping genes in each group of DEGs.

Group	Key TF	Overlapped genes	*p-*value	List of overlapped genes
ESP/Control	Nfkb1	17	0.000	Ptgs2, Mmp3, Tnfrsf25, Nos2, Ppargc1a, Slc1a2, Ccnd3, Cxcl1, Cxcl2, Per1, Dmp1, Egr2, Atp12a, Ccl2, Hes1, Il6, Lcn2
	Rela	10	0.000	Mmp3, Hes1, Ptgs2, Cxcr4, Nos2, Ccl2, Atp12a, Il6, Slc1a2, Dmp1
	Jun	10	0.000	Ccnd3, Ptgs2, Cxcl1, Mmp3, Tnfsf11, Nos2, Fosl1, Ccl2, Cxcl2, Il6
	Sp1	9	0.003	Slc7a5, Egr1, Ccnd3, Ccl2, Il6, Atp12a, Tnfsf11, Ptgs2, Col2a1
	Trp53	8	0.001	Ptgs2, Pmp2, Btg2, Klf2, Agtr1a, Col2a1, Plk3, Egr1
	Runx2	6	0.000	Col2a1, Tnfsf11, Panx3, Hes1, Axin2, Tg
	Ep300	6	0.000	Col2a1, Axin2, Flt4, Ptgs2, Il6, Nos2
	Cebpb	5	0.000	Ptgs2, Il6, Btg2, Cxcr4, Ppargc1a
	Foxo1	5	0.000	Ppargc1a, Egr1, Gck, Klf2, Ccl2
	Nr3c1	4	0.000	Npas4, Ccnd3, Ptgs2, Hes1
HCF/Control	Nfkb1	15	0.000	Mmp3, Nos2, Slc1a2, Cxcl1, Ankrd2, Sod2, Dmp1, Bcl2a1d, Selp, Il23a, Atp12a, Cd38, Ccl2, Il6, Lcn2
	Rela	12	0.000	Cd38, Sod2, Mmp3, Selp, Nos2, Ccl2, Atp12a, Bcl2a1d, Il6, Slc1a2, Dmp1, Il23a
	Jun	9	0.000	Prdm1, Cxcl1, Mmp3, Tnfsf11, Penk, Nos2, Il23a, Ccl2, Il6
	Sp1	8	0.010	Csrp2, Ccl2, Il6, Sod2, Atp12a, Tnfsf11, Col2a1, Kcnn3
	Rel	5	0.000	Sod2, Nos2, Il23a, Ccl2, Bcl2a1d
	Ep300	5	0.000	Col2a1, Flt4, Il6, Mt1, Nos2
	Stat3	5	0.002	Nos2, Prdm1, Mt1, Il6, Il23a
	Nfe2l2	4	0.003	Krt16, Tnfsf11, Sod2, Mt1
	Fos	4	0.003	Mt1, Il6, Nos2, Penk
	Runx2	4	0.003	Col2a1, Tnfsf11, Panx3, Ibsp
pll/Control	Sp1	16	0.001	Myh11, Fos, Bglap2, Cd55, Egr1, Ccnd3, Abca1, Il6, Hmgcr, Ptgs2, Tal1, Socs3, Proc, Cadm1, Nrgn, Gfap
	Trp53	15	0.000	Ptgs2, Ccnb2, Fos, Uhrf1, Lif, Gap43, Btg2, Foxm1, Ccnb1, Ccna2, Krt19, Ankrd1, Dusp1, Plk3, Egr1
	Nfkb1	15	0.000	Ptgs2, Nos2, Ppargc1a, Ccnd3, Lif, Edn1, Fos, Ccl20, Dusp1, Egr2, Cd38, Junb, Nfkbiz, Hmgcr, Il6
	Jun	11	0.000	Hmgcr, Nefl, Ccnd3, Ptgs2, Fos, Penk, Nos2, Fosl1, Il6, Socs3, Gfap
	Fos	10	0.000	Bdnf, Socs3, Nefl, Tinagl1, Fos, Il6, Egr1, Nos2, Penk, Hmgcr
	Stat3	9	0.000	Nos2, Ccnd3, Gfap, Il6, Socs3, Ccl20, Egr1, Fos, Lif
	Ep300	8	0.000	Fos, Ptgs2, Ccnb2, Il6, Edn1, Mef2c, Nos2, Socs3
	Cebpb	7	0.000	Ptgs2, Il6, Btg2, Cxcr4, Ppargc1a, Socs3, Fos
	Rela	7	0.027	Cd38, Fos, Ptgs2, Cxcr4, Ccl20, Nos2, Il6
	Creb1	6	0.000	Fos, Noct, Ppargc1a, Nfkbiz, Penk, Ptgs2

### Migration and cytokine expression alterations in BMSCs

**Figure 7:**
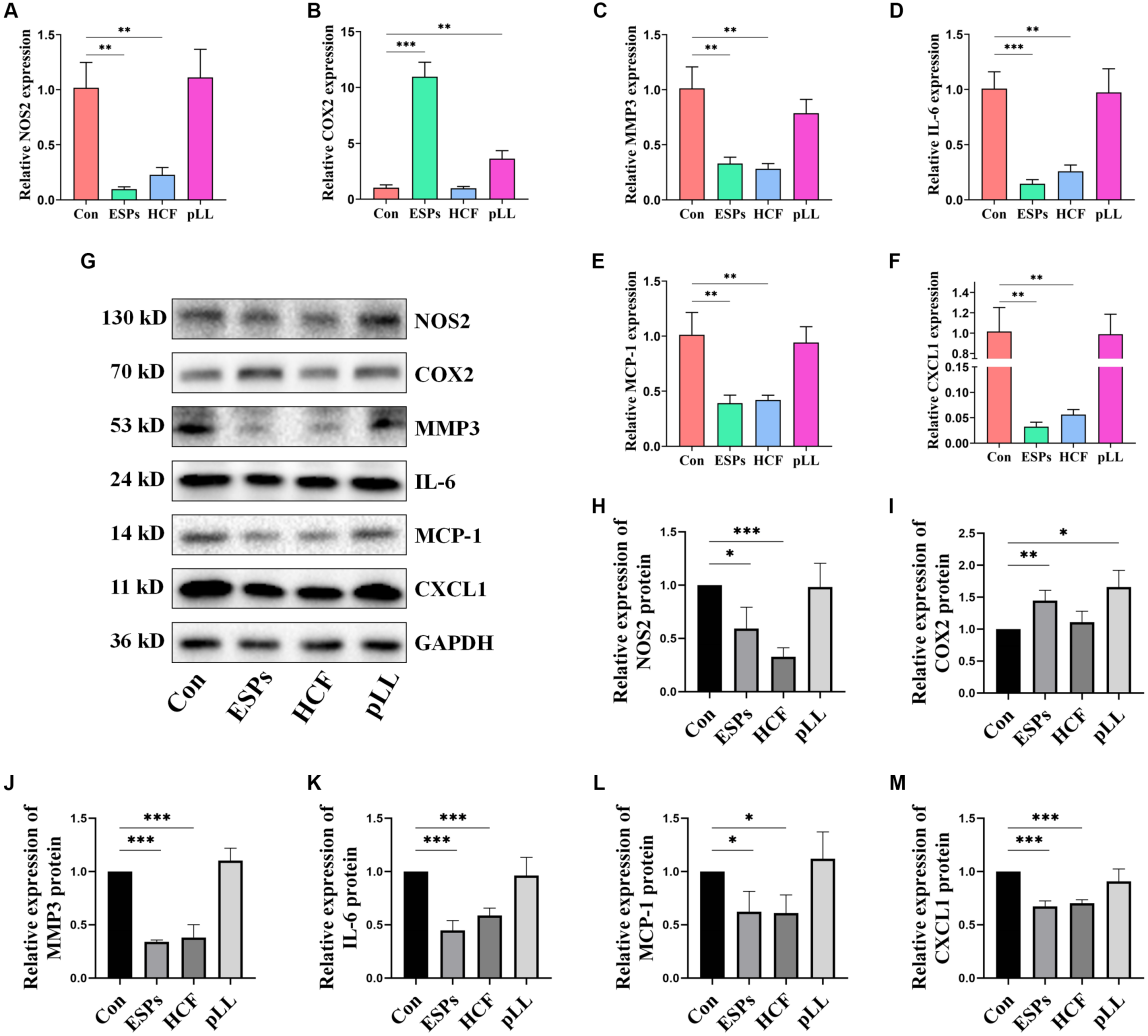
Validation of expression of relevant genes in cells at mRNA and protein levels using qRT-PCR and Western blots. **(A)** NOS2 mRNA expression in three groups of BMSCs after antigen stimulation. **(B)** COX2 mRNA expression in three groups of BMSCs after antigen stimulation. **(C)** MMP3 mRNA expression in three groups of BMSCs after antigen stimulation. **(D)** IL-6 mRNA expression in three groups of BMSCs after antigen stimulation. **(E)** MCP-1 mRNA expression in three groups of BMSCs after antigen stimulation. **(F)** CXCL1 mRNA expression in three groups of BMSCs after antigen stimulation. **(G)** Protein expression levels of NOS2, COX2, MMP3, IL-6, MCP-1, and CXCL1 in the BMSCs cells after antigen treatment. **(H)** Protein expression statistics of NOS2. **(I)** Protein expression statistics of COX2. **(J)** Protein expression statistics of MMP3. **(K)** Protein expression statistics of IL-6. **(L)** Protein expression statistics of MCP-1. **(M)** Protein expression statistics of CXCL1. The data are presented as the mean ± SD. *p < 0.05, **p < 0.01, ***p < 0.001, significantly different from control group.

The scratch assay revealed that all hydatid antigens significantly reduced BMSCs migration, indicating a potential mechanism by which the parasite evades host immune responses (Figures 2E,F). qRT-PCR and Western blot analyses showed downregulation of MCP-1, CXCL1, NOS2, IL-6, and MMP3, alongside an upregulation of COX2 in the ESPs and pLL groups, suggesting a shift towards an anti-inflammatory phenotype in BMSCs (Figure 7).

### Changes in cytokine concentrations in cell supernatants

**Figure 8:**
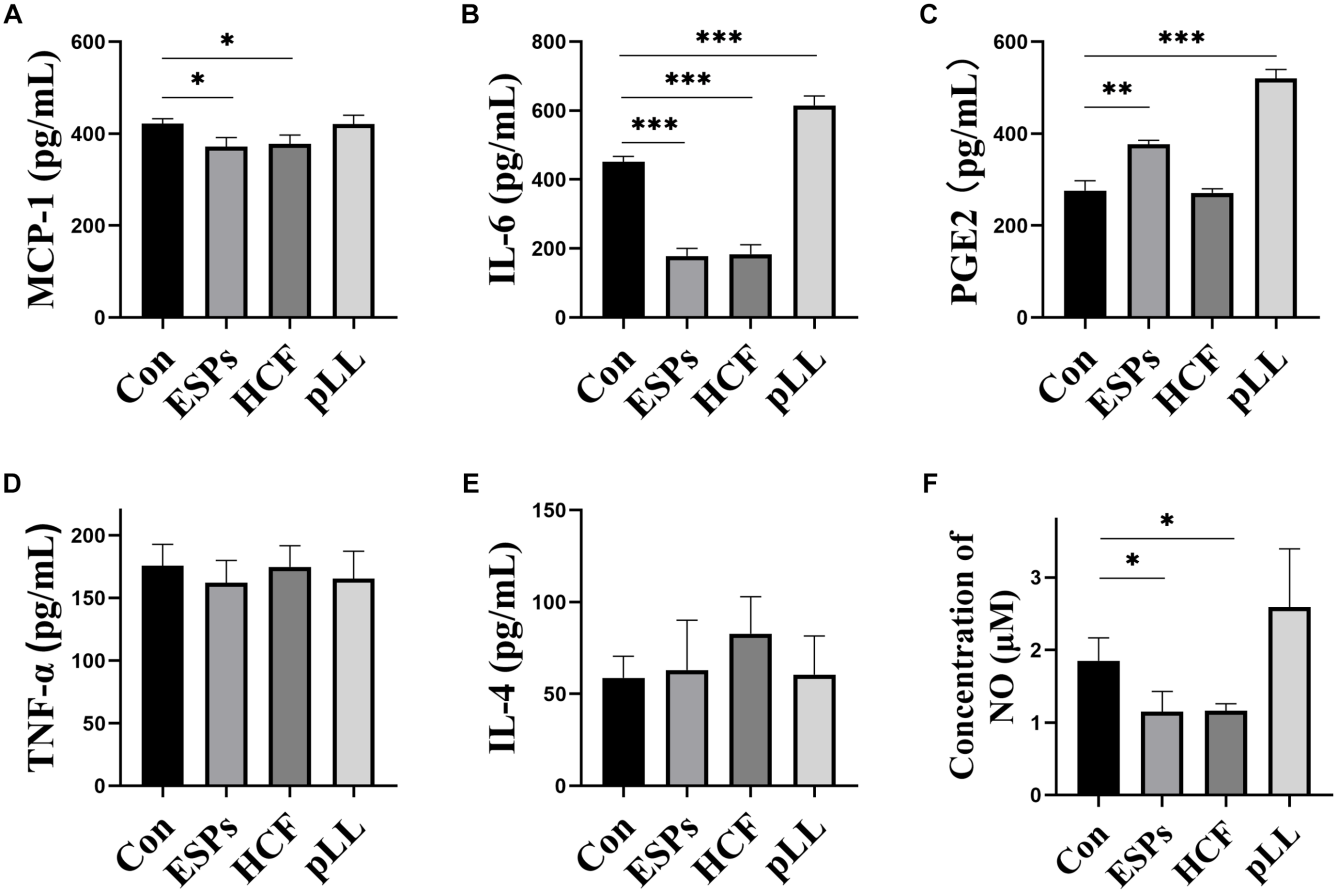
Determination of cytokines and NO content in cell supernatants using ELISA and assay kits. **(A)** Protein expression statistics of MCP-1 in the cell supernatant. **(B)** Protein expression statistics of IL-6 in the cell supernatant. **(C)** Protein expression statistics of PGE2 in the cell supernatant. **(D)** Protein expression statistics of TNF-α in the cell supernatant. **(E)** IL-4 expression statistics in cell supernatants. **(F)** NO concentration statistics in cell supernatants. The data are presented as the mean ± SD. *p < 0.05, **p < 0.01, ***p < 0.001, significantly different from control group.

ELISA results indicated significant reductions in MCP-1 and IL-6 in ESPs and HCF groups, with elevated PGE2 in ESPs and pLL groups. No significant alterations were observed for TNF-α and IL-4 (Figures 8A–E), suggesting that echinococcal antigen may decrease the expression of chemotactic factors and some pro-inflammatory factors, but not entirely in the conventional sense of anti-inflammatory/pro-inflammatory cytokines.

### NO concentration changes in cell supernatants

A significant decrease in NO content was noted in the ESPs and HCF groups post 48-h antigen exposure, with no changes in the pLL group (Figure 8F), suggesting the potential for echinococcal antigen to exert regulatory effects through mechanisms outside of cytokine signaling pathways.

## Discussion

Parasitic infections pose significant health challenges, particularly in underdeveloped regions. Hydatid disease, characterized by a wide range of infections and severe consequences, has gained significant attention. The disease occurs when *Echinococcus granulosus* eggs are accidentally ingested by intermediate hosts, including humans. The eggs release oncospheres in the host’s intestinal tract, which then enter the systemic vascular system via the portal vein and eventually colonize various sites in the body (Gottstein et al., 2014). Skeletal hydatid disease, though relatively rare, is a serious form of the disease. Patients typically experience local pain, bone destruction, nerve compression, and pathological fractures around the affected area. Surgical and pharmacological treatments have limitations, making complete eradication of the lesions challenging and leading to catastrophic impacts on patients’ ability to work and overall health (Steinmetz et al., 2014).

Considering the transvascular route of hydatid disease infection *in vivo*, we hypothesized that tissue cells surrounding blood vessels in parenchymal organs, such as MSCs, may be exposed to hydatid antigens over an extended period (Crisan et al., 2014; Murray et al., 2014). Bone tissue, rich in MSCs and immune cell types, led us to propose that hydatid antigens can exert important regulatory functions through MSCs during the course of bone encysted disease.

Our experiments used three crude antigen types, all of which were found to interact with the host’s internal environment in previous studies. This was evident from the presence of characteristic antigenic species in secretions and vesicular fluids of PSCs circulating in patients’ blood, as well as the direct contact between immune cells and the laminated layer (Casaravilla et al., 2014; Pan et al., 2014, 2017; Mohammed et al., 2018). We observed significant differences in gene expression in BMSCs co-cultured with the three antigens compared to the control group, indicating that BMSCs can sense and respond to different hydatid antigens. Notably, ESPs showed a higher proportion of overlapping differentially expressed genes (DEGs) with HCF, suggesting similar regulatory effects. This aligns with the expectation that HCF contains excretory secretion products of PSCs. On the other hand, pLL, with its distinct chemical composition and tissue distribution, exhibited distinct regulatory patterns.

The differential expression of cytokines is particularly noteworthy as it plays a crucial role in the paracrine regulatory capacity of MSCs. Chemokines, pro-inflammatory cytokines, and anti-inflammatory cytokines are among these cytokines. Pathway enrichment analysis revealed significant differences in the expression levels of chemokines, such as MCP1, CXCL1, and CXCL5, following intervention with ESPs and HCF.

To validate our findings, we conducted sequencing and confirmed a decrease in the mRNA and protein expression of certain chemokines, highlighting the physiological significance of chemokine expression in MSCs. Chemokines are known to regulate immune cell recruitment, including monocytes and neutrophils, which are typically the first to be recruited around the parasite (Legler and Thelen, 2016). Neutrophils exhibit anti-parasitic effects through phagocytosis, degranulation, and the formation of neutrophil extracellular trapping networks (NETs) (Díaz-Godínez and Carrero, 2019; Omar and Abdelal, 2023). Similarly, macrophages may hinder parasite survival through phagocytosis, NO production, and pro-inflammatory cytokine release (Silva-Álvarez et al., 2016; Lin et al., 2021). Therefore, the downregulation of chemokine expression in MSCs induced by parasite antigens may facilitate the innate immune evasion of parasites. Additionally, certain chemokines, such as CCL2, have been implicated in regulating the recruitment of MSC-osteoprogenitor cells and protecting against apoptosis induced by the external environment (Yao et al., 2021; Zhang et al., 2023). Thus, alterations in chemokine expression in MSCs can have multiple biological effects and are closely associated with lesion development.

Our sequencing and experimental results validate that exposure of BMSCs to ESPs and HCF antigens leads to a decrease in the expression of NOS2/NO and IL-6. NOS2 facilitates the conversion of arginine to NO, which directly eliminates parasites and significantly impacts their survival through immunomodulatory mechanisms such as the differentiation of Th1- and Th17-type cells and T cell infiltration (James, 1995). Previous studies have suggested that as hydatid disease progresses, there appears to be a tendency to induce a low NOS2-expressing phenotype in host immune cells like macrophages, T cells, and B cells (Steers et al., 2001; Soufli et al., 2015). Our experiments demonstrate that this phenomenon also manifests in MSCs.

Interestingly, protein interaction network analysis indicated that the cytokine IL-6 ranked highest in association among all three groups of DEGs after antigenic intervention. This suggests that IL-6 likely plays a crucial role in the immunomodulatory effects of MSCs induced by hydatid antigens. The role of IL-6 in parasite immunomodulation is multifaceted and controversial. On one hand, IL-6 has been implicated in promoting macrophage differentiation towards the M2 subtype, which is more favorable for chronic parasite infection and increases host susceptibility (Philipp et al., 2018). On the other hand, high expression of IL-6 may have a positive impact on the recruitment, migration, and survival of monocytes, macrophages, MSCs, and neutrophils (Wen et al., 2021). The low expression of IL-6 and NO in MSCs indicates a tendency for anti-inflammatory secretion. However, this tendency appears to be limited, as we did not observe differences in the concentration of more typical pro−/anti-inflammatory cytokines, such as TNF-α or IL-4, in the cell supernatants.

Furthermore, our sequencing and experimental results confirm that the antigens ESPs and pLL significantly enhance the expression of COX2/PGE2 in MSCs. PGE2 plays a crucial role in the immunomodulatory function of MSCs, including promoting the differentiation of immune cells towards anti-inflammatory and reparative phenotypes while inhibiting the activation of T cells and the secretion of pro-inflammatory cytokines (Duffy et al., 2011; English, 2013). Therefore, the upregulation of COX2/PGE2 expression indicates the transformation of MSCs into a phenotype that possesses anti-inflammatory regulatory capabilities (Kulesza et al., 2022). This discovery highlights the potential mechanism by which hydatid antigens exert their regulatory influence on host adaptive immunity.

Furthermore, based on TF gene prediction, Nfkb1(p50) is identified as a major transcription factor that exhibits significant differences in the three antigens compared to controls, forming part of the NF-κB dimer. Activation of NF-κB is closely associated with the expression of various chemokines and cytokines (Tanaka et al., 2014; Chadha and Chadee, 2021). Studies conducted on other hydatid disease foci have also shown high expression of Nfkb1 around the foci, suggesting its involvement in the establishment of parasite immune evasion (Tilioua et al., 2020). Hence, the NF-κB signaling pathway may play a crucial role in mediating the immune effects of hydatid antigens.

The protein distribution between certain groups in gel electrophoresis appears similar, mainly due to the dominance of high-concentration fetal bovine serum over lower-concentration antigen proteins. Fetal bovine serum plays a crucial role in preserving low-concentration proteins and maintaining consistency with the protein concentration in the control group. Our study observed a significant decrease in the migration capacity of MSCs following all three antigenic stimuli. Additionally, we found that both the ESPs and HCF groups exhibited decreased expression of matrix metalloproteinase 3 (MMP3), which plays a role in breaking down various types of proteins, such as collagen and fibronectin, facilitating cell movement through the extracellular matrix (Assis-Ribas et al., 2018; Chang et al., 2021). However, it remains uncertain whether the altered cell migration ability is directly linked to MMP3 expression, as the pLL group, which did not show significant changes in expression, also demonstrated reduced cell migration ability. Further investigation into the role of MSC migration and matrix metalloproteinase expression in parasitic infections may yield intriguing findings.

Lastly, significant enrichments related to cellular metabolism, such as steroid and carbohydrate metabolism, as well as oxidoreductase activity, were consistently observed in all sequencing results. Prior studies have suggested that *Echinococcus granulosus* infection may exert immunomodulatory effects by influencing the oxidoreductase class of host immune cells (Wang et al., 2019). The metabolism of MSCs plays a crucial role in cellular differentiation and immune function. For instance, MSCs exhibit a preference for glycolysis during proliferation but shift towards oxidative phosphorylation-based metabolism during osteoblastic and lipogenic differentiation (Denu and Hematti, 2016; Reichert et al., 2021). To induce immune polarization, MSCs require a metabolic shift towards aerobic glycolysis (Rahimi et al., 2021; Zhuang et al., 2021). This intriguing information suggests the possibility that hydatid antigens exert biological effects by altering cellular energy metabolism.

However, it is important to acknowledge certain limitations of our study. Hydatid lesions, as confined solid foci, display an antigenic distribution influenced by spatial distance, with varying concentrations of the same antigen in different extracellular environments. Therefore, our interventions were based on the cell proliferation assay results, ensuring the antigen concentration did not impact cellular activity. In future research, it is essential to consider the regulatory roles of other active factors, such as complement components, in cellular functions to gain a comprehensive understanding of the interactions involved. The complexity of protein types and non-protein metabolites in hydatid crude antigens presents challenges in deciphering the specific pathways of action for different antigens. Given that the modulatory effect of antigens likely arises from multiple antigenic responses, the selected antigenic species in our study are believed to partially replicate the specific microenvironment of MSCs in hydatid disease, offering a foundational point and supporting evidence for further mechanistic investigations.

## Conclusion

Our study elucidates the biological impact of hydatid disease antigens on BMSCs, indicating that these antigens can induce a significant shift in MSC cytokine profiles and migration patterns, favoring an anti-inflammatory secretion phenotype. The upregulation of COX2/PGE2 and downregulation of chemokines and pro-inflammatory cytokines, such as IL-6 and NOS2/NO, suggest that MSCs may play a role in the immunomodulatory mechanisms of *Echinococcus granulosus* to evade host immune responses. The decrease in MSC migration and changes in gene expression related to cellular metabolism highlight the potential for hydatid antigens to alter cellular energy metabolism and contribute to the persistence of infection. These findings enhance our understanding of the pathophysiology of hydatid disease and may guide the development of novel therapeutic approaches targeting the interaction between MSCs and hydatid antigens. Further research is warranted to dissect the specific molecular pathways involved and their implications for disease progression and treatment.

## Data availability statement

The data presented in the study are deposited in the GEO database, accession number GSE256257.

## Ethics statement

The animal study was approved by laboratory animal ethics committee of first affiliated hospital of Xinjiang Medical University. The study was conducted in accordance with the local legislation and institutional requirements.

## Author contributions

XW: Writing – review & editing, Writing – original draft, Supervision, Software, Resources, Methodology, Funding acquisition, Data curation. WM: Writing – review & editing, Investigation, Formal analysis. ZY: Writing – review & editing, Project administration, Investigation. QJ: Writing – review & editing, Resources, Methodology, Funding acquisition. JM: Writing – review & editing, Resources, Investigation. ZX: Investigation, Project administration, Resources, Software, Supervision, Validation, Visualization, Writing – original draft, Writing – review & editing.
