# Supplementary figures and images for: Immunomodulatory effects of hydatid antigens on mesenchymal stem cells: gene expression alterations and functional consequences
Source: Front Microbiol. 2024 Apr 9;15:1381401. doi: 10.3389/fmicb.2024.1381401 (PMC11035891; doi:10.3389/fmicb.2024.1381401)

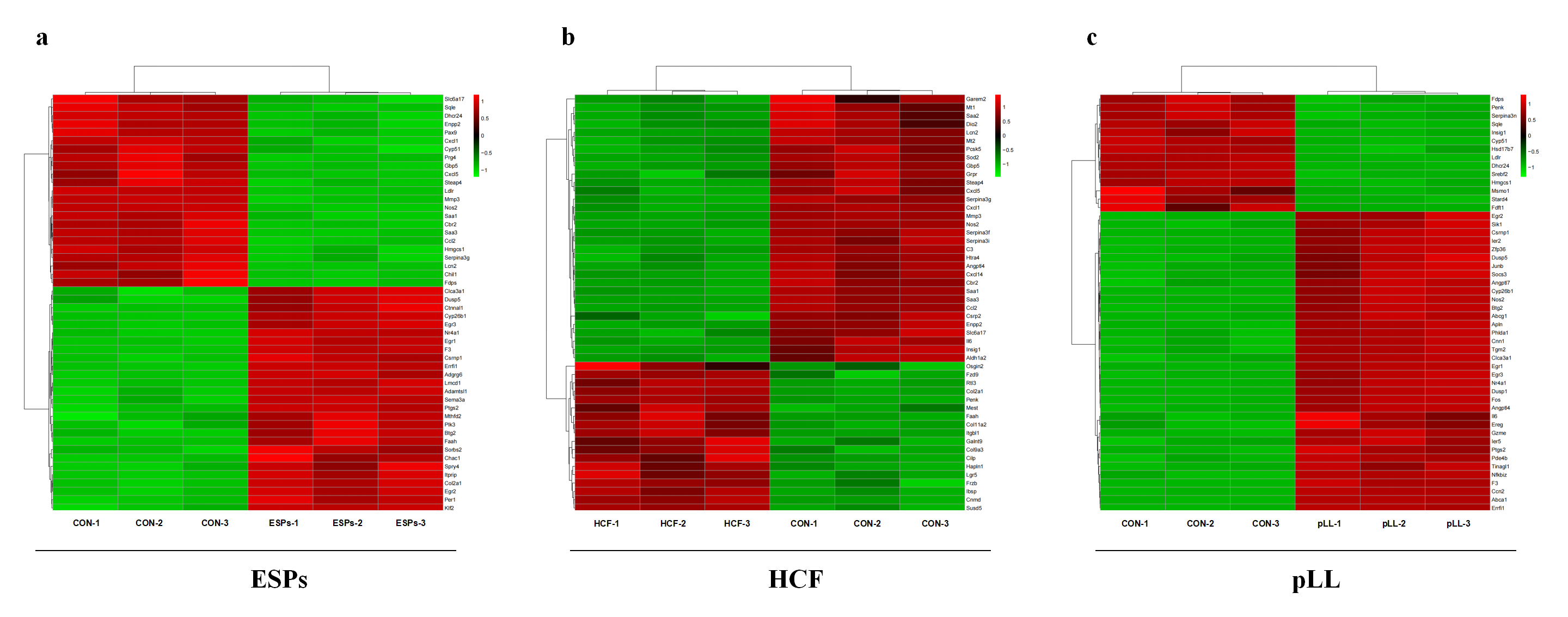

Supplement: Supplementary file 1 [file Image_1.TIF]

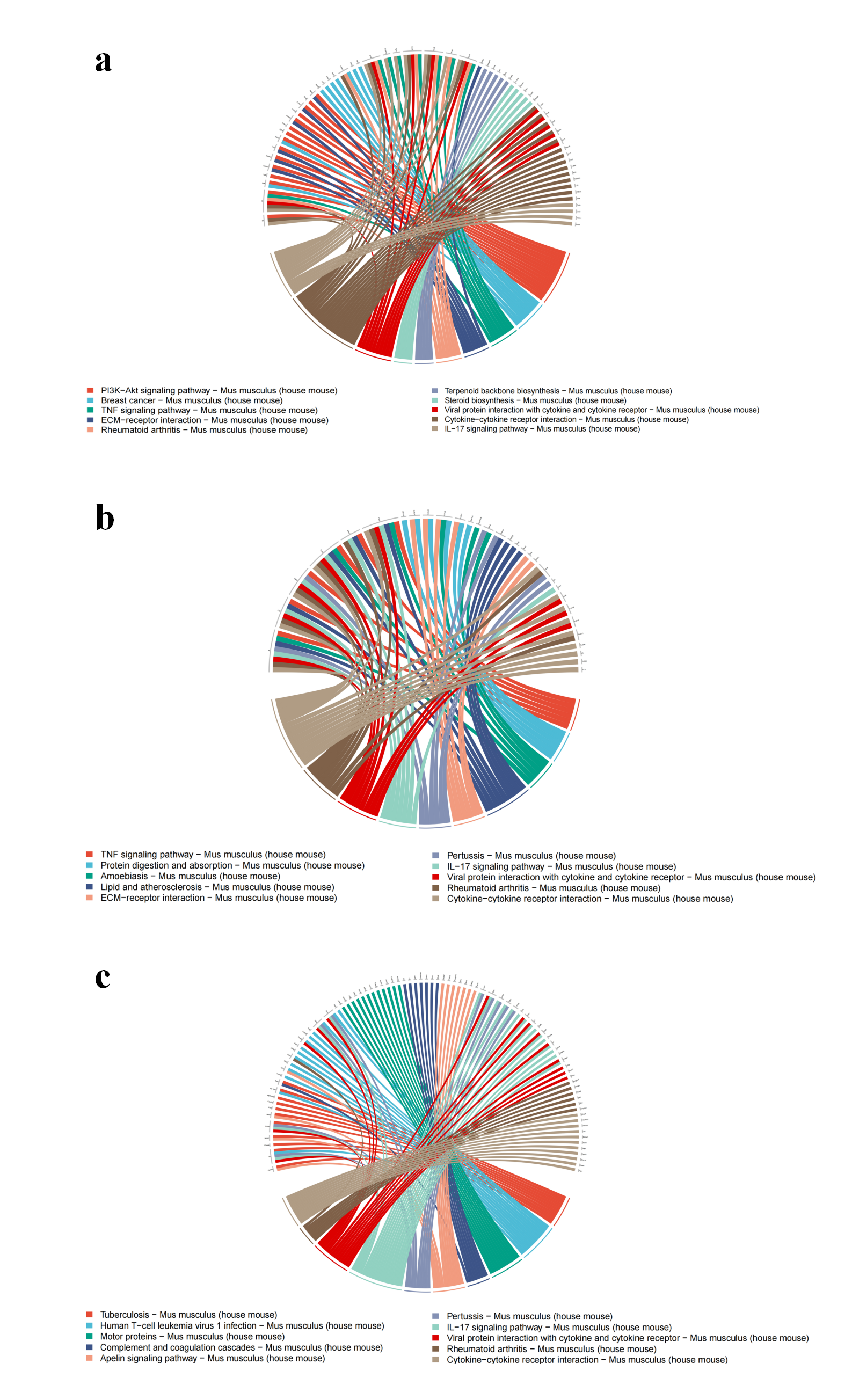

Supplement: Supplementary file 2 [file Image_2.TIF]

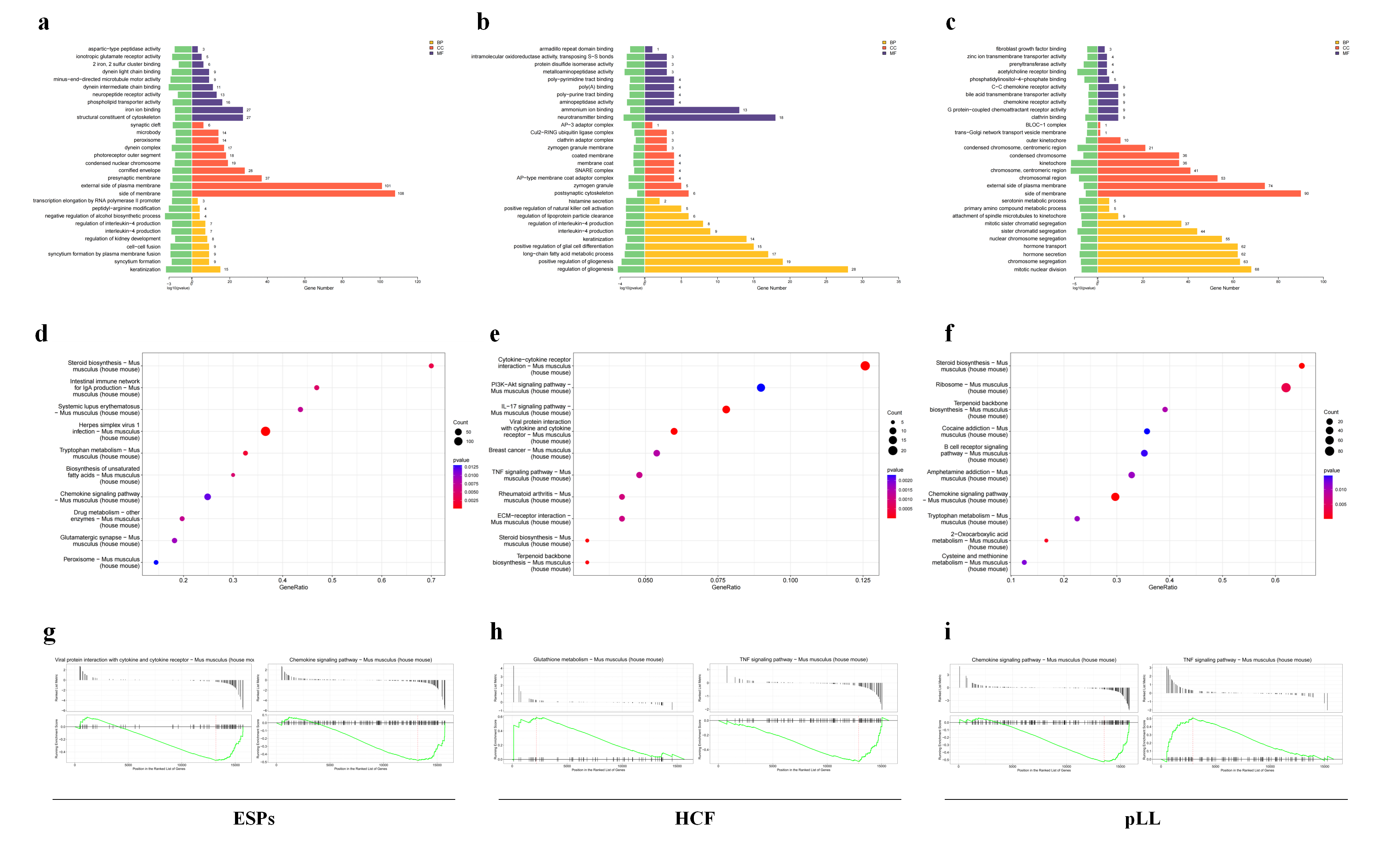

Supplement: Supplementary file 3 [file Image_3.TIF]

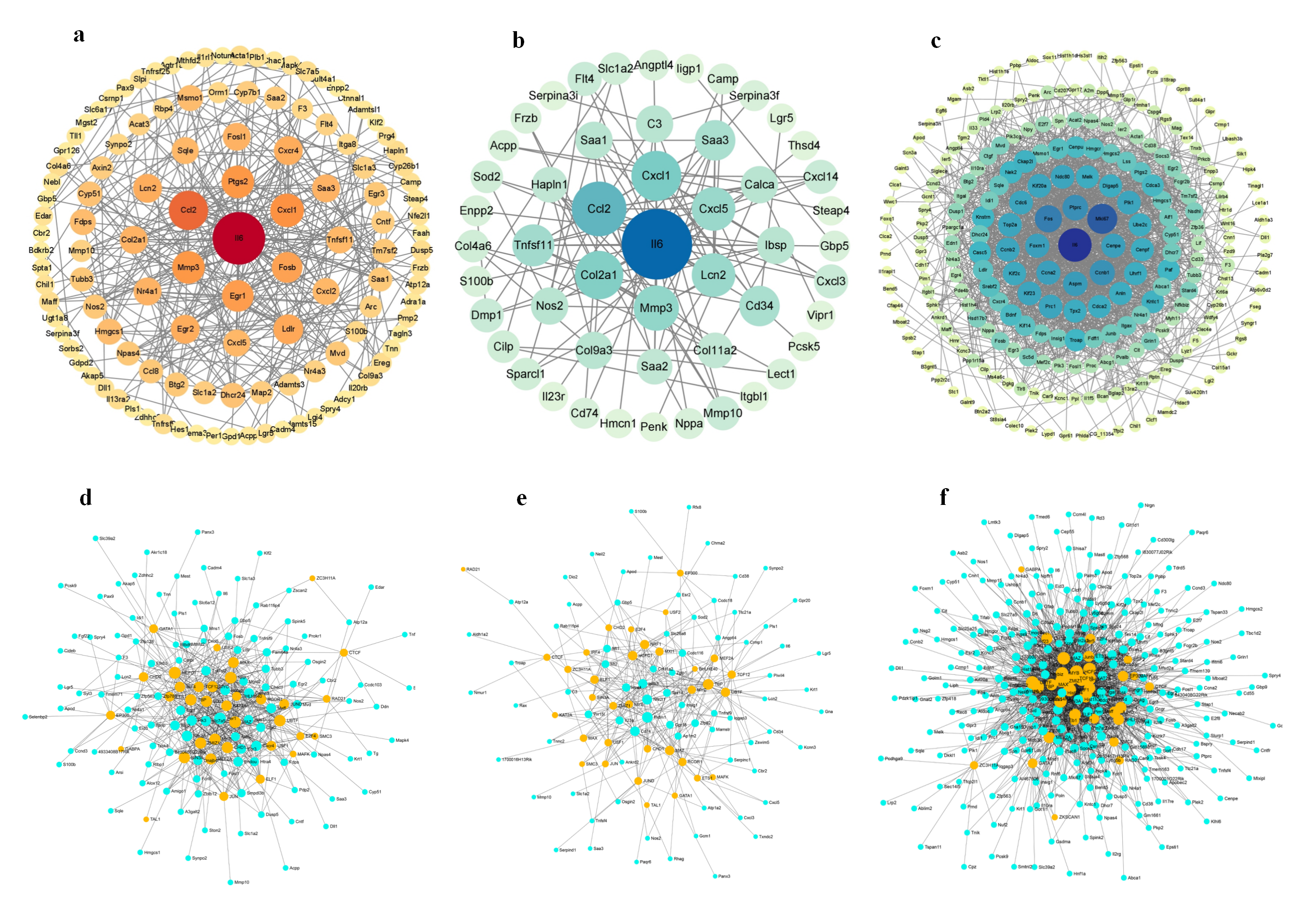

Supplement: Supplementary file 4 [file Image_4.TIF]
